# Replacing water and nutrients for ethanol production by ARTP derived biogas slurry tolerant *Zymomonas mobilis* strain

**DOI:** 10.1186/s13068-019-1463-2

**Published:** 2019-05-20

**Authors:** Guowei Duan, Bo Wu, Han Qin, Weiting Wang, Qiong Tan, Yonghua Dai, Yao Qin, Furong Tan, Guoquan Hu, Mingxiong He

**Affiliations:** 1 Biomass Energy Technology Research Centre, Key Laboratory of Development and Application of Rural Renewable Energy (Ministry of Agriculture and Rural Affairs) Biogas Institute of Ministry of Agriculture and Rural Affairs, Section 4-13, Renmin Rd. South, Chengdu, 610041 People’s Republic of China; 2 Graduate School of Chinese Academy of Agricultural Science, Beijing, 100081 People’s Republic of China; 3 College of Pharmacy and Biological Engineering, Chengdu University, Chengdu, 610041 People’s Republic of China

**Keywords:** Ethanol production, Biogas slurry, *Zymomonas mobilis*, ARTP mutagenesis, Adaptive laboratory evolution

## Abstract

**Background:**

Reducing fresh water consumption and nutrient addition will be an effective way to reduce the whole cost of bioethanol production. On the other hand, treatment of biogas slurry derived from anaerobic digestion (AD), in which a great amount of nutrients is still left, costs too much to remove these pollutants. It would be beneficial for both digestate valorization and ethanol production if biogas slurry is used for producing bioethanol. However, both hyperosmosis and potential biotoxic components of the biogas slurry can severely inhibit fermentation.

**Results:**

In this study, two rounds of atmospheric and room temperature plasma (ARTP) mutagenesis combined with adaptive laboratory evolution (ALE) were applied to improve the adaptability and genetic stability of *Zymomonas mobilis* in biogas slurry. Mutants D95 and S912 were identified. Growth of the mutants was remarkably improved in biogas slurry. The highest ethanol productivity reached 0.63 g/L/h which was 61.7% higher than ZM4 (0.39 g/L/h). Genomic re-sequencing results also revealed that single nucleic variations (SNVs) and Indels occurred in the mutants, which are likely related to inhibitor in biogas slurry and low pH tolerance.

**Conclusions:**

Our study demonstrated that these mutant strains have great potential to produce ethanol using biogas slurry to replace fresh water and nutrients.

**Electronic supplementary material:**

The online version of this article (10.1186/s13068-019-1463-2) contains supplementary material, which is available to authorized users.

## Background

Over the last decade, the ecological crisis and energy dilemma are becoming increasingly serious, which stimulated worldwide research on the use of renewable resources for fuel production [1]. Bioethanol as one of the green energy resources was an alternative fuel for relieving energy issues [2]. With growth of the bioethanol industry in European Union (EU), United States of America (USA) and China, different strategies of bioethanol production from agriculture residues have been established [3–5]. Especially, producing ethanol from inexpensive substrates was conducted. For example, producing ethanol from lignocellulosic biomass is seen as a way to reduce the cost and carbon emissions during ethanol production [1, 6]. Up to now, the consumption of water resource and nutrients in production process are still major costs during large-scale production. Nearly 1.9–9.8 L of water are consumed for producing 1 l of cellulosic ethanol [7]. Besides, industrial nutrients used for cellulosic ethanol production included steep corn liquor (CSL) and diammonium phosphate (DAP) would cost $1.7 to $2.2 million per year in a 200 million liters ethanol plant [8]. Therefore, finding cheaper alternative nutrient sources would play a vital role in promoting the development of ethanol industry.

Anaerobic digestion (AD) is the primary method to treat different types of organic wastes [9]. With the growth of the global energy demands, biogas from wastes, residues, and energy crops play a vital role in solving energy dilemma by providing an effective pattern of not only solving fuel shortages, but avoiding the risks of wastes being released directly into the environment [10]. The production of biogas through anaerobic digestion has aroused public concern, especially in the European Union (EU) [11] and China [12]. With the volume of digestate expanding substantially, biogas slurry from large scale biogas plants is also increasing rapidly in large livestock or poultry farms. The risks of nutrient pollution caused by improper utilization and disposal of biogas slurry are major environmental concern with the land application of biogas slurry [9]. The disposal of biogas slurry has become a major bottle-neck in the development of the biogas industry, especially for the livestock and poultry breeding industry [13]. Furthermore, the majority of the nitrogen, ammonia, phosphate, potassium and other nutrients essential for bacteria growth remain in the biogas residue and slurry after AD, and several studies show that nutrients in biogas slurry are richer than raw manure counterparts [14, 15]. It is reported that biogas slurry used as culture medium in culturing yeast and microalgae would significantly reduce the cost of production and improve the productivity [5, 16]. Thus, the application of biogas slurry in ethanol production is considered a new opportunity for green process. Our previous study also showed that the use of biogas slurry as an alternative to process water and nitrogen sources may decrease the cost of cellulosic ethanol production by 10.0–20.0% [17].

*Zymomonas mobilis* is an attractive ethanologenic candidate with desirable industrial characteristics [18]. *Z. mobilis* possesses better environmental adaptability, higher ethanol productivity and ethanol tolerance than many yeasts [19]. Via the unique Entner–Doudoroff (ED) pathway, *Z. mobilis* generates less ATP and microbial biomass while converting many carbon sources in glycolytic metabolism, which results in the higher glucose metabolic flux three to five times that of *S. cerevisiae* [20]. Several kinds of bio-based products that are produced by *Z. mobilis* have emerged, while the weaknesses of narrow substrate utilization and sensitivity to acetic acid have prevented its commercial development. Therefore, many strategies have been developed to improve *Z. mobilis* strains with desirable features, such as improving inhibitor-tolerance via random mutation and widening utilization capability of substrates via genome modification [21, 22]. Although it is feasible and cost effective for using biogas slurry in cellulosic ethanol production, only 56.3 kg of ethanol was produced by *Z. mobilis* through fermentation of 1000 kg of dried corn straw. We attempted to find an effective way to improve the productivity and adaptability of *Z. mobilis* in biogas slurry.

Random mutagenesis has been demonstrated as a powerful strategy to enhance tolerances in *Z. mobilis* [22]. Compared with traditional genetic engineering, atmospheric and room temperature plasma (ARTP) has unique advantages which is a powerful mutagenesis technology for biobreeding in recent years [23]. It has been proved that ARTP is effective in improving production efficiency and enhancing robustness for many species, such as bacteria, fungi, and plants [24–26]. Although ARTP is powerful, no desirable mutant possibly could be obtained under high selective pressure, such as high acetic acid level or low pH value, if only one single round of ARTP mutagenesis was carried out. Recently, the strategy combining ARTP mutagenesis with adaptive laboratory evolution (ALE) has been successfully applied in improving cell growth and succinic acid production efficiency of *Escherichia coli* [27] and *Z. mobilis* [21], demonstrating it as an efficient strategy to obtaining stable mutant strains.

So, in this study, two rounds of breeding of ARTP mutagenesis combined with adaptive laboratory evolution (ALE) were applied to improve the adaptability and genetic stability of *Z. mobilis* in biogas slurry. Mutant strain S912 was obtained which has better adaptability and higher ethanol productivity in biogas slurry. Furthermore, only carbon source was added to the biogas slurry for ethanol fermentation, which greatly reduced the nutrition cost. The process conditions were optimized to achieve higher ethanol titer and ethanol productivity, and used in fed-batch fermentation.

## Results and discussion

### Mutation and screening of the mutants

Biogas slurry has been documented as a potential source of nutrients for cellulosic ethanol production by *Z. mobilis* associated with pretreatment [17]. However, previous studies had also indicated that the inhibition on *Z. mobilis* growth was observed when it was cultivated in biogas slurry. To achieve higher biomass and ethanol production, two rounds of the breeding that ARTP mutagenesis combined with ten rounds of ALE were applied to improve the adaptability and genetic stability of *Z. mobilis* in undiluted biogas slurry (shown in Additional file 1: Figure S1). After the first-round breeding, five mutants D91, D92, D95, D161, and D172 that tolerated undiluted biogas slurry were obtained. After the first ARTP mutagenesis, dozens of mutant strains and wild type strain ZM4 were subjected to the ten rounds of the ALE procedure, and mutants D91, D92, D95, D161, and D172 successfully survived while the wild type ZM4 failed to survive in the process.

**Fig. 1:**
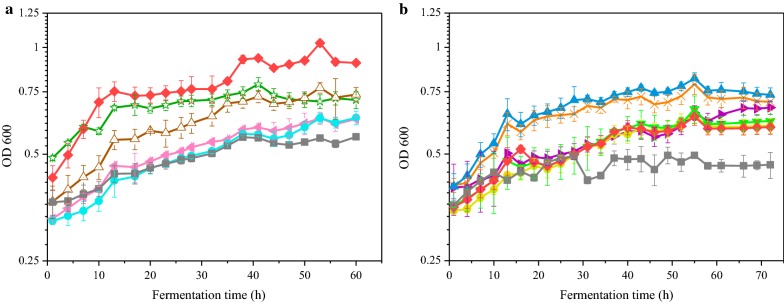
**a** Growth of *Zymomonas mobilis* mutant strains in biogas slurry medium at pH 6.0 which were obtained in 1st-round breeding. The *Z. mobilis* strains were as follows: D91 (Olive star), D92 (Brown up-triangle), D95 (Red diamond), D161 (Pink left-triangle), D172 (Light blue circle), ZM4 (control) (Gray square). **b** Growth of *Z. mobilis* mutant strains in biogas slurry medium at pH 3.8 which were obtained in 2nd-round breeding. The *Z. mobilis* strains were as follows: S1 (Purple right-triangle), S9 (Yellow circle), S10 (Orange cross). S11 (Green down-triangle), S912 (Blue up-triangle), D95 (control) (Red diamond), ZM4 (control) (Gray square). Growth was determined by measuring OD_600_ value. The cultivations were carried out by automated turbidimeter with 10% inoculum at 30 °C. The biogas slurry with 50 g/L glucose was sterilized using a 0.22 μm filter membrane. All cultivations were carried out in triplicate. Error bars show standard deviation (n = 3)

The growth curves of mutant strains and ZM4 were detected by automated turbidimeter at 30 °C without shaking. As shown in Fig. 1a, the five mutants were inoculated in biogas slurry medium with 50.0 g/L glucose at pH 6.0. The maximum OD_600_ values of the five mutant strains were increased by 12% to 62% compared with the wild type strain (Fig. 1a). Possessing greater potential to grow faster than other strains in undiluted biogas slurry, D95 was chosen for the second-round breeding.

Low pH circumstances can effectively prevent microbial contamination during fermentation, and thus can realize the fermentation under non-aseptic conditions [28]. Considering its strong adaptability in undiluted biogas slurry as well as low pH value, D95 that achieved the highest biomass was chosen for another round of breeding to select mutants against low pH value. After the second ARTP mutagenesis, dozens of mutant strains, D95 and wild type strain ZM4 were subjected to the ten rounds of the ALE procedure, and five mutants S1, S9, S10, S11 and S912 that were able to tolerate biogas slurry at pH 3.8 survived. As shown in Fig. 1b, the five mutants, D95 and ZM4 were inoculated in biogas slurry medium with 50.0 g/L glucose at pH 3.8. The growth curves of strains were detected by automated turbidimeter at 30 °C without shaking. The growth curve shows that S912 grew much faster than others in biogas slurry medium with low pH value. OD_600_ values of S912 were, respectively, increased by 23% and 58% compared with the parental strain D95 and wild type strain ZM4. After two rounds of breeding, ten mutant strains were identified, of which S912 showed stronger tolerance to high concentration of biogas slurry and low pH.

### Enhanced adaptability of *Z. mobilis* in biogas slurry

**Fig. 2:**
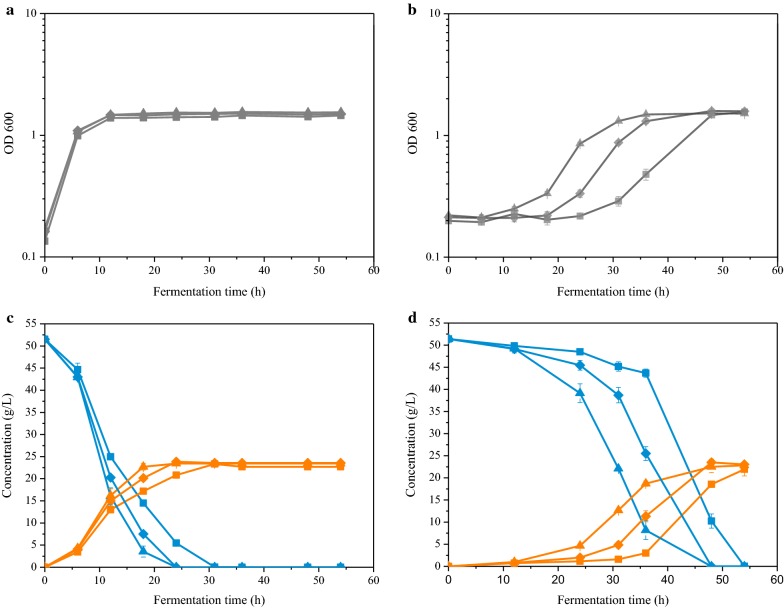
Batch fermentation by *Z. mobilis* at 30 °C and pH 6.0. Growth of the strains (Gray line) in **a** rich medium (RM) and **b** biogas slurry medium. The consumed glucose (Blue line) and produced ethanol (Orange line) during fermentation in **c** RM and **d** biogas slurry medium are shown. The *Z. mobilis* strains were as follows: ZM4 (Square), D95 (Diamond), S912 (Up-triangle). Biogas slurry was sterilized using a 0.22 μm filter membrane. The 10% (v/v) inoculum cells were used for all fermentations. All fermentations were conducted in 150-mL flask contains 100 mL medium with 50 g/L glucose. All fermentations were carried out in triplicate. Error bars show standard deviation (n = 3)

After two rounds of breeding, the growth and fermentation performance of mutant strains D95 and S912 in biogas slurry medium with 50.0 g/L glucose at pH 6.0 were further evaluated. The evaluated fermentation was carried out with 10% (v/v) inoculum in 100 mL of rich medium (RM) or biogas slurry medium at 30 °C without shaking. As shown in Fig. 2a, c, the growth in rich medium (RM) without the stress was no difference in growth and fermentation performance between D95 and S912. The ethanol productivities of D95 and S912 were 0.98 g/L/h, which is 31% higher than that of wild type strain ZM4.

As shown in Fig. 2b, d, the growth and ethanol productivity of mutant strains D95 and S912 were remarkably improved in biogas slurry medium. The ethanol productivity of S912 was 0.63 g/L/h, which is 37% and 62% higher than D95 and ZM4, respectively (Additional file 1: Table S2). The ethanol yield of S912 reached 88% of theoretical yield, four percentage points higher than that of D95 and S912 (Additional file 1: Table S2).

### Optimized fermentation medium

Nutrients, such as compounds of carbon, nitrogen, and phosphorus (C, N, P), are important for sustenance of various life forms. The optimization of fermentation parameters is a prior necessity as it plays an essential part in the success of a bioprocess industry. The pH values during fermentation, the concentrations of various initial glucose concentrations, PO_4_^3−^-P, NH_4_^+^-N of biogas slurry, and were evaluated before fed-batch fermentation. The cells of S912 were inoculated in four group 100-mL biogas slurry mediums, which were listed in Table 1 with 10% (v/v) inoculum, and grown at 30 °C without shaking.

**Table 1:** Fermentative medium

Group^a^	pH	Glucose (g/L)	KH_2_PO_4_/H_3_PO_4_ (g/L)	NH_4_^+^-N(mg/L)
a	3.5–9.5	50	–	–
b	6.0	50–175	–	–
c	6.0	50	1	–
d	6.0	50	–	178–1911

^a^Medium for different experimental groups. The medium for determining optimal (a) pH value, (b) initial concentration of glucose, (c) adding of KH_2_PO_4_ or H_3_PO_4_, (d) concentration of NH_4_^+^-N

**Fig. 3:**
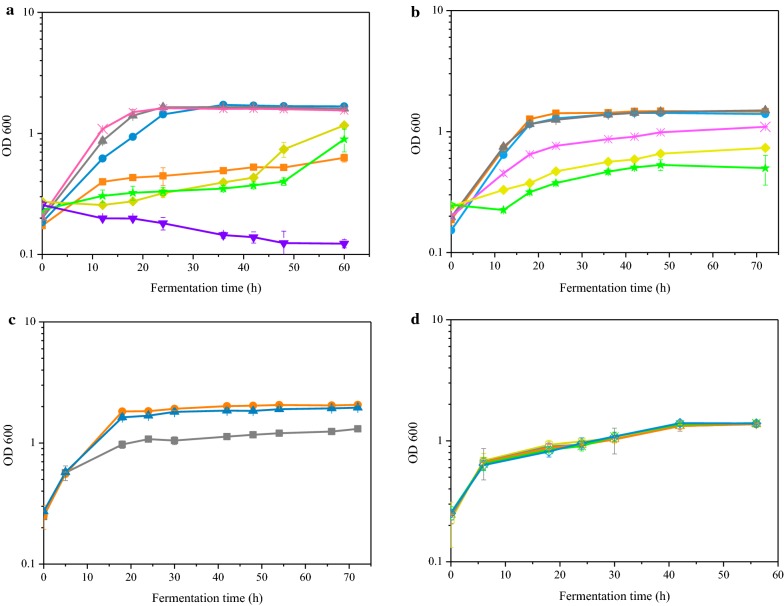
Growth of *Z. mobilis* strain S912 in different biogas slurry mediums which are listed in Table 1. **a** The growth curves of S912 in the biogas slurry medium with different pH values as follows: pH 3.5 (Orange square), pH 4.5 (Blue circle), pH 5.5 (Gray up-triangle), pH 6.5 (Pink cross), pH 7.5 (Yellow diamond), pH 8.5 (Green star), pH 9.5 (Purple down-triangle). **b** The growth curves of S912 in the biogas slurry medium with different initial glucose concentrations as follows: 50 g/L (Orange square), 75 g/L (Blue circle), 100 g/L (Gray up-triangle), 125 g/L (Pink cross), 150 g/L (Yellow diamond), 175 g/L (Green star). **c** The growth curves of S912 in the biogas slurry medium with 1.0 g/L KH_2_PO_4_ (Blue up-triangle), 1.0 g/L H_3_PO_4_ (Orange circle), or without addition (Gray square). **d** The growth curves of S912 in the biogas slurry medium with different initial NH^4+^-N concentrations as follows: 178 ± 2 mg/L (Yellow square), 647 ± 4 mg/L (Gray circle), 1059 ± 8 mg/L (Orange triangle), 1487 ± 1 mg/L (Green cross), 1911 ± 6 mg/L (Blue diamond). Growth was determined by measuring OD_600_ value. The 10% (v/v) inoculum cells were used for all batch cultivations. The biogas slurry was sterilized using a 0.22 μm filter membrane. All fermentations were conducted in 150-mL flask contains 100-mL biogas slurry medium at 30 °C without shaking. All fermentations were carried out in triplicate. Error bars show standard deviation (n = 3)

The pH value of biogas slurry generally ranged from 6.7 to 9.2 [29] that was not in the optimal range of *Z. mobilis* [30]. Besides, the previous experiments showed a decreasing pH value with the increasing of cell growth. If glucose was added to the medium repeatedly, the pH value would continue to decrease before the stationary phase which would significantly inhibit microbial fermentation efficiency. It was necessary to control the pH value of the medium within a reasonable range during the fermentation process. In group a, the effect of the pH value during fermentation was examined at a pH range of 3.5–9.5. As shown in Fig. 3a, the cell growth was not inhibited at a pH range of 4.5–6.5. While an apparent inhibition of cell growth was observed when the initial pH reduced from 4.5 to 3.5 or raised from 6.5 to 7.5 which indicated that the appropriate pH values ranged from 4.5 to 6.5. The result provides a reference for the application of biogas slurry during fermentation. Before fermentation, the pH value of biogas slurry medium should not exceed 6.5. During open fermentation, the pH value of biogas slurry medium should not be lower than 3.5. The decrease of pH value in medium was probably caused by the byproducts of *Z. mobilis* such as CO_2_, acetic acid, lactic acid, etc. [31].

In group b, the initial glucose concentration was adjusted between 50 g/L and 200 g/L (Fig. 3b), the results showed a decreasing cell growth of S912 with the increasing initial glucose concentration. Compared with the initial glucose concentration under 100 g/L, the cell density (OD_600_) under 125 g/L glucose was decreased by 44% at 18 h which indicated that glucose concentration higher than 100 g/L generated negative impacts on cell growth. Compared with the previous studies, the maximum initial glucose concentration of *Z. mobilis* in biogas slurry was much lower than that in RM and food waste hydrolysate [3]. On the one hand, excessive sugar concentration in the medium would product negative effects on the growth of *Z. mobilis*. On the other hand, the high osmotic pressure caused by concentrated sugar medium and complex compounds in biogas slurry generated inhibitors to microorganism. This result indicated that the tolerance of S912 in high osmotic pressure medium was also significantly improved.

The concentration of PO_4_^3−^-P in biogas slurry generally ranged from 0.2 mg/L to 231 mg/L. Compared with synthetic medium, the concentration of PO_4_^3−^ -P in biogas slurry was much less. In group c, the addition of 1 g/L H_3_PO_4_ and 1 g/L KH_2_PO_4_ could significantly improve the cell density (OD_600_) which increased by 50% and 58%, respectively (Fig. 3c). Furthermore, the initial pH value of biogas slurry could be adjusted to optimal range for *Z. mobilis* by adding H_3_PO_4_. The addition of 1 g/L H_3_PO_4_ in biogas slurry medium could improve the fermentation efficiency and adjust the pH value.

In group d, the cell growth of S912 was examined with the concentration of NH_4_^+^-N in biogas slurry medium ranging from 178 ± 2 mg/L to 1911 ± 6 mg/L. As shown in Fig. 3d, there was no obvious difference in cell density of *Z. mobilis* in biogas slurry with various concentration of NH_4_^+^-N which was obtained from the same biogas plant. The concentration of NH_4_^+^-N in biogas slurry ranged from 178 ± 2 mg/L to 1911 ± 6 mg/L which could meet the needs of the growth of *Z. mobilis* completely. These results also indicated that accumulation of NH_4_^+^-N in the biogas slurry might not cause inhibition of ammonia on S912.

### Fed-batch fermentation by mutant strain S912

The ethanol productivity of S912 was stable after passing ten generations in biogas slurry. To study the possibility of industrial scale ethanol production from biogas slurry by biogas slurry tolerant strain S912, fed-batch fermentation was conducted in 5-L bioreactor at pH 6.0 and pH 3.8, respectively. The 3-L optimal biogas slurry medium (100 g/L glucose and 1 g/L H_3_PO_4_) was used in all fed-batch cultivations with 10% (v/v) inoculum and grown at 30 °C without agitation.

**Fig. 4:**
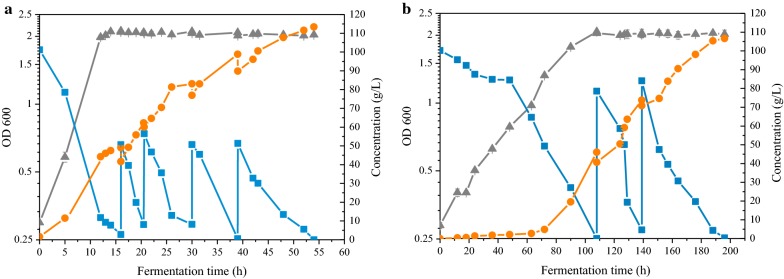
Fed-batch fermentation by S912 in 5-L bioreactor. Growth (Gray triangle), consumed glucose (Blue square) and produced ethanol (Orange circle) in **a** sterilized biogas slurry medium at pH 6.0; **b** unsterilized biogas slurry medium at pH 3.8 are shown. Growth was determined by measuring OD_600_ value. The biogas slurry used in sterilized fermentation was sterilized using a 0.22 μm filter membrane. The biogas slurry used in unsterilized fermentation was centrifuged at 4000 rpm and 4 °C, and collected supernatant. The biogas slurry mediums contain 100 g/L glucose and 1 g/L H_3_PO_4_. All fed-batch fermentations were carried out with 10% (v/v) inoculum cells at 30 °C without agitation. The working volume of fed-batch fermentation was 3 L, and the increasing of pH value was an indicator for replenishing with glucose until the final working volumes reached 4.0 L. All fed-batch fermentations were carried out in triplicate. Error bars show standard deviation (n = 3)

The results show that ethanol titer and productivity were remarkably improved in biogas slurry medium. In fed-batch fermentation at pH 6.0, biomass was continuous and relatively stable before 16 h (Fig. 4a). The ethanol titer reached the maximal value of 113.4 g/L at 54 h. After the second fed–batch, the maximum ethanol productivities and ethanol yields of theoretical were 4.13 g/L/h and 96% (Additional file 1: Table S3), respectively, which was more than 321% and 4% higher than that obtained in RM at pH 6.0 (Additional file 1: Table S2). The results implied the feasibility of ethanol fermentation in optimal biogas slurry medium by S912.

It is reported that ethanol production under non-sterilized condition can save 30–40% energy consumption in cooking starch and sterilization during ethanol production. Hence, the fed-batch fermentation was conducted openly condition (without sterilization at pH 3.8) to determine the feasibility of open fermentation. The ethanol titer of fed-batch fermentation was significantly increased which reached the maximal value of 107.6 g/L at 194 h (Fig. 4b). The maximum ethanol productivity was 1.07 g/L/h, which show 70% higher than that obtained in RM at pH 3.8 (Additional file 1: Table S3).

In this study, the ethanol titers increased by 32% (sterilized) and 25% (unsterilized) compared with yeast fermented in anaerobic digestate effluent (ADE) [5], respectively. Showing high ethanol productivity and stable fermentation performance in biogas slurry medium, mutant strains S912 possesses potential for industrial application. Furthermore, after open fermentation, the turbidity, the concentration of NH_4_^+^-N in biogas slurry decreased with the cultivation of *Z. mobilis* which hinted that it is of significance to combine the disposal of biogas slurry with production of ethanol. Our study further verified that replacing water and nutrients with biogas slurry for ethanol production was an environmental-friendly and economical process for ethanol production.

### Genome re-sequencing of mutants

To determine the underlying genetic determinants responsible for mutant strains enhanced adaptability and ethanol productivity in biogas slurry, mutant genomes were sequenced to investigate genetic changes caused by ARTP mutagenesis. All mutation sites are listed in Tables 2 and 3. The position of the mutation sites in genome are shown in Additional file 1: Figure S2.

**Table 2:** Mutation sites in CDS

Locus	Mutation	AA Change	1st	2nd	Gene	Product
849,208	C → T	Syn.	+	+	*ZMO_RS03765*	Arginine-tRNA ligase
849,311	C → A	P505H	+	+		
971,059	T → A	Syn.	+	+	*ZMO_RS09160*	IS5 family transposase
971,308	A → G	U71Q	+	+	*ZMO_RS09165*	IS5 family transposase
971,332	T → C	I63V	+	−		
51,967	C → T	G276R	−	+	*ZMO_RS00235*	Glutamine–fructose-6-phosphate aminotransferase
590,452	G → A	E432K	−	+	*ZMO_RS02620*	DNA repair protein RadA
1,657,469	Delete CAGTTTC	Shift	+	+	*ZMO_RS07255*	Carbamoyl phosphate synthase large subunit

Syn.: Synonymous, variation in nucleotide led to no amino acid change. +/− indicate the presence/absence of variation in the genome, respectively

**Table 3:** Mutation sites in intergenic regions

Locus	Mutation	1st	2nd	Gene	Product
975,503	T → G	+	+	*ZMO_RS04290/ZMO_RS04295*	Monofunctional biosynthetic peptidoglycan/cytochrome c
975,506	G → A	+	+		
975,509	C → T	+	+		
975,523	C → T	+	+		
975,525	A → T	+	+		
975,528	T → G	+	+		
975,532	A → T	+	+		
975,537	A → C	+	+		
975,540	G → T	+	+		
975,547	T → G	+	+		
1,612,575	G → A	+	+	*ZMO_RS07065/ZMO_RS07070*	alpha/beta hydrolase/tRNA-Met
2,055,763	T → C	+	+	*ZMO_RS09095/END*	uroporphyrinogen decarboxylase/END
1,448,818	Delete C	−	+	*ZMO_RS06410/* *ZMO_RS06415*	FUSC family protein/DNA polymerase III subunit delta

**Fig. 5:**
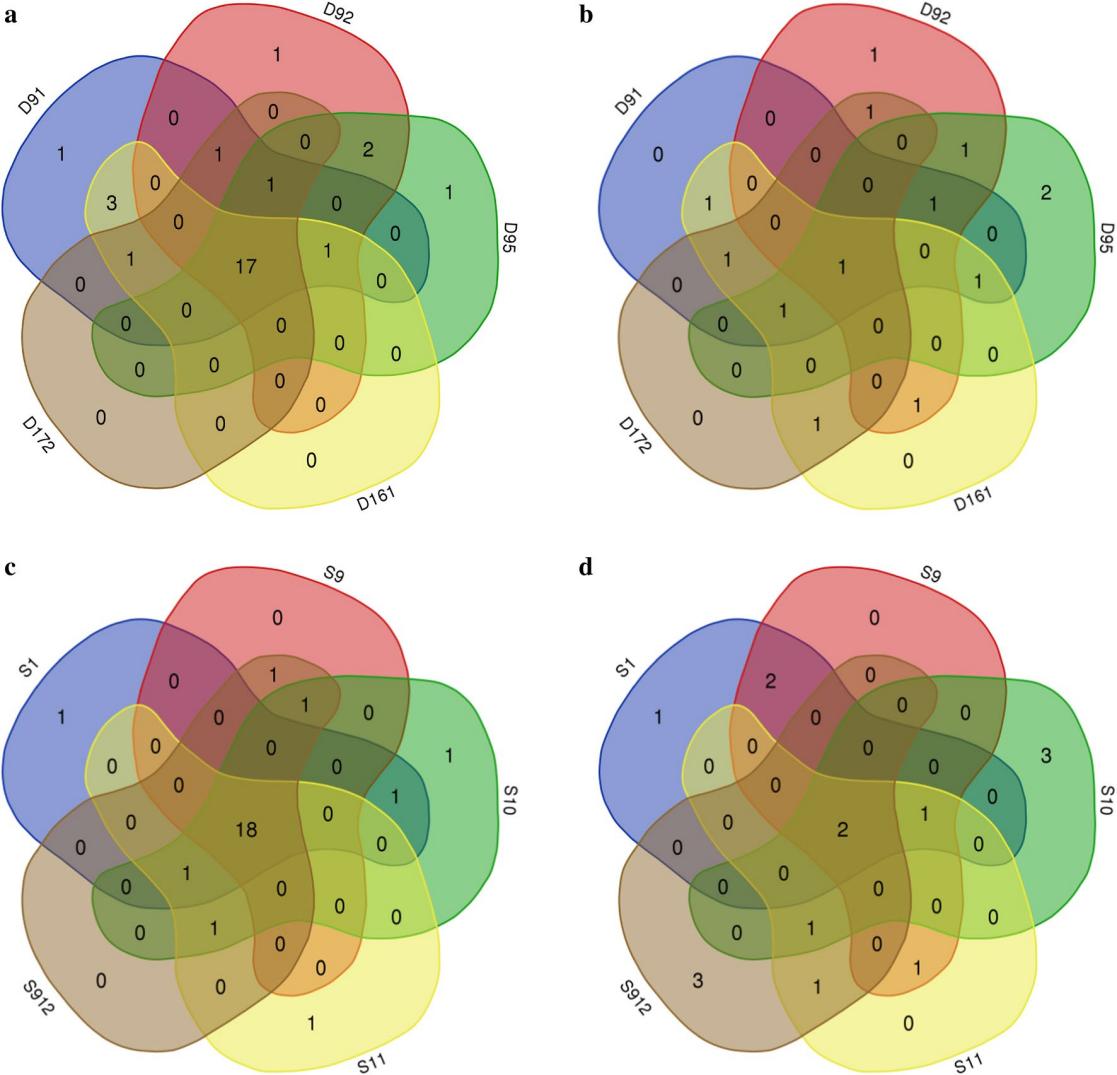
Venn grams of SNVs and Indels in CDSs after ARTP mutagenesis. **a** SNVs of first-round mutants; **b** Indels of first-round mutants; **c** SNVs of second-round mutants; **d** Indels of second-round mutants

As shown in Fig. 5a, b, seventeen single nucleotide variations (SNVs) were detected in all mutants obtained from the first round of mutagenesis-five of which were located in coding sequence (CDS) and twelve in intergenic regions and one deletion. Five SNVs in CDS regions located in three different genes, including two synonymous mutations located in gene *ZMO_RS09160* and *ZMO_RS03765*, one non-synonymous mutation located in gene *ZMO_RS03765* which encodes arginine-tRNA ligase, and two non-synonymous mutations located in gene *ZMO_RS09165* which encode IS5 family transposase. Besides, seven nucleotides deletion occurred in CDS of gene *ZMO_RS07255* which encodes carbamoyl-phosphate synthase large subunit. The role of carbamoyl-phosphate synthase in the carbon and nitrogen metabolism cycles is very important. The carbamoyl-phosphate synthase contains an ammonia tunnel and a carbamate tunnel which connect the three distinct active sites, as conduits between unstable reaction intermediates (ammonia and carbamate) and successive active sites [32]. It was reported that the mutation in carbamoyl-phosphate synthase large subunit could affect bacterial utilization of several carbon and nitrogen resources in minimal medium MMX and extracellular enzyme activities [33]. And the large subunit of carbamoyl-phosphate synthase has two homologous carboxy phosphate domains, both of which possess ATP-binding sites [32], and it could provide enough ATP to exclude intracellular protons produced by the low pH of the acid [34].

After the second round of mutagenesis, eighteen SNVs and two indels were detected (Fig. 5c, d). The variation loci of the 2^nd^-round mutants (Additional file 1: Figure S2b) were quite similar with the 1^st^-round mutants (Additional file 1: Figure S2a) but two distinct non-synonymous SNVs were found in glutamine–fructose-6-phosphate transaminase and DNA repair protein RadA which was encoded by *ZMO_RS00235* and *ZMO_RS02620*, respectively, and new single nucleotide deletion occurred between gene *ZMO*-*_RS06410* and *ZMO_RS06415.* It has been reported that glutamine–fructose-6-phosphate aminotransferase is critical for cells against organic acid stress [35, 36]. DNA repair protein RadA involved in homologous recombination and DNA damage repair processes of many organisms [37, 38] which facilitated DNA repair in *E. coli* cells damaged by UV radiation, X-rays, and chemical agents [39].

In addition, ten identical SNVs occurred in intergenic regions between *ZMO_RS04290* and *ZMO_RS04295* which encode monofunctional biosynthetic peptidoglycan and cytochrome c, respectively. The reason of successive mutations occurred in the intergenic regions was unknown. Several genes which was detected SNVs in the intergenic region might be involved in improve adaptability of *Z. mobilis* in biogas slurry. The alpha/beta hydrolase coded by *ZMO_RS07065* has a Nucleophile-His-Acid catalytic triad to adapt to a complex physical environment which evolved to efficiently operate on medium with different chemical composition or physicochemical properties [40]. FusC family protein coded by *ZMO_RS06410* was related to improve fusidic acid resistance [41] and methicillin-resistant [42], which may benefits for *Z. mobilis* to survive in biogas slurry.

## Materials and methods

### Biogas slurry collection and pretreatment

Biogas slurry used in this study was collected from a storage pond after treatment in an anaerobic continuous stirred tank reactor (CSTR) which was provided by a biogas plant located in Sichuan province in China. They were immediately transported to the laboratory and stored at 4 °C. Original biogas slurry was centrifuged at 4000 rpm and 4 °C. The supernatant was collected and used in unsterilized fermentation. Then the collected supernatant sterilized by filtrated with 0.22 μm filter membrane, which was used in sterilized fermentation.

### Microorganisms and growth media

*Zymomonas mobilis* wild type strain ZM4 was served as parental strain for mutation. The glycerol stocks of ZM4 generated by ARTP mutagenesis was grown at 30 °C and maintained on an agar rich medium (RM) containing 50.0 g/L glucose, 10 g/L yeast extract, 2.0 g/L KH_2_PO_4_, and 1.5–2.0% (w/v) agar. A single colony was inoculated in 10 mL of RM and grown overnight at 30 °C without shaking. The cell pellets were harvested by centrifuged at 3000 rpm and 4 °C and then inoculated in 100 mL of RM or biogas slurry medium in 150-mL flask and grown at 30 °C without shaking. Before inoculation, the glucose was added to biogas slurry at a concentration of 50 g/L, and pH value was adjusted to 6.0 or 3.8. Biogas slurry mediums used in optimization experiments are listed in Table 1. All growth and fermentations were carried out in triplicate.

### Atmospheric and room temperature plasma(ARTP)mutagenesis and adaptive laboratory evolution (ALE)

The strategy of ARTP mutagenesis compared with ALE was described in Additional file 1: Figure S1). In the first-round breeding, ZM4 was cultured as a parental strain of ARTP mutagenesis to select biogas slurry tolerant strains. One milliliter of fresh overnight ZM4 cultures containing 10^6^–10^8^ cells was centrifuged at 3000 rpm and 4 °C, followed by suspension in the same volume of 0.9% NaCl solution. Ten microliters of the suspension were treated using Type M ARTP Mutagenesis Biobreeding Machine (Wuxi Tmaxtree Biotechnology Co., Ltd., China) for 30 s or 45 s. Gas helium (He) of high purity served as the working gas at the flow rate of 10–20 standard liter per minute (SLM). The power input was set at 100 or 120 V, and the ARTP jet temperatures was 22 °C. The treated cells were recovered by suspended in 1 mL of RM for 6 h. Then 50.0–100.0 μL of the suspended cultures were spread on sterilized biogas slurry with1.5–2.0% (w/v) agar (50.0 g/L glucose, pH 6.0), and grown until colonies formed.

Secondly, dozens of clones were improved in ALE. The selected colonies were inoculated into a new 15-mL Falcon tube containing 5 mL of biogas slurry medium (50.0 g/L glucose, pH 6.0), and then cultured into the same medium by the method of serial batch transfer (i.e., performed by a tube-to-tube transfer of the inoculums into the new media), repeated ten times. After this process, the surviving clones were selected. Then the growth curves of mutant strains were detected by automated turbidimeter (Bioscreen C, Labsystem, France). And the best strain was selected as parental strain for the second mutagenesis.

The second mutagenesis was executed to select low pH-tolerant mutants. The ALE and screening strategy were the same as the first-round breeding. All of cultivations were performed at 30 °C without shaking.

### Determination of pH value, the concentration of initial glucose, PO_4_^3−^-P, and NH_4_^+^-N

To determine optimal medium for the growth of *Z. mobilis* in biogas slurry, four group batch experiments were performed mediums of which are listed in Table 1. The S912 was inoculated in 10 mL of RM and grown overnight at 30 °C without shaking. The cell pellets were harvested by centrifuged at 3000 rpm and 4 °C and then inoculated in 100 mL biogas slurry medium (Table 1) in 150-mL flask. All of cells were grown at 30 °C without shaking. All experiments were carried out in triplicate.

### Fed-batch fermentation in a 5-L bioreactor

The fed-batch fermentations were carried out in 5-L fermentors (BIOSTAT^®^ B plus, Sartorius stedim), with 3 L of biogas slurry medium. The experiments were conducted at pH 6.0 (sterilized) and pH 3.8 (without sterilization), respectively. Cultivation methods were the same as in batch operation. 10% (v/v) inoculum cells were transferred with final working volumes of 3.0 L. The S912 cells were pre-grown for 12 h in RM and harvested from 300 mL of RM cultures by centrifuging at 3000 rpm and 4 °C, then inoculated in 3 L of biogas slurry medium which contains 100 g/L glucose and 1 g/L H_3_PO_4_ (pH 6.0 or 3.8). The pH of the culture was recorded constantly and automatically regulated at 6.0 and 3.8, respectively, with 5 M NaOH during fermentation. The cultivation was carried out at 30 °C without agitation. The increasing of pH value was an indicator for replenishing with glucose until the final working volumes reached 4.0 L. Samples were collected to follow the cells growth, glucose and ethanol concentrations. All fermentations were carried out in triplicate.

### Analysis methods

#### Analysis nutrient components of biogas slurry

After filtration with a 0.22 μm membrane and dilution by 50 times, the concentrations of NH_4_^+^-N and NO_3_^−^-N in fermentation medium were measured by AA-3 autoanalyzer (Bran+ Luebbe, Germany). Chemical oxygen demand (COD) was determined according to standard methods described by APHA. Besides, inductively coupled plasma optical emission spectroscopy (ICP-OES; PlasmaQuant PQ9000, Germany) was used to analyze other nutrient components. The data are shown in Additional file 1: Table S1.

#### Glucose, ethanol, and cells determination

After filtration with a 0.22 μm membrane and dilution by 10 times, the concentrations of glucose and ethanol in fermentation medium were analyzed by high performance liquid chromatography (HPLC; Agilent 1200) with an HPX-87H ion exclusion column (Bio-Rad Aminex) which was used at 35 °C with 5 mM H_2_SO_4_ as the mobile phase [38]. The cell density was determined by a spectrophotometer detector (Jingke UV765, Shanghai) at wavelength 600 nm.

#### Genomic DNA isolation and Genome re-sequencing

The genomic DNAs (gDNAs) of *Z. mobilis* extraction were performed by a Bacterial DNA Kit (Omega Biotek, USA). The quality of gDNAs was detected by Qubit 3 Fluorometer (ThermoFisher, USA), and then checked by gel electrophoresis (0.7% agarose, 120 V/cm, 50 min). PCR amplification of bacterial 16S rRNA gene was performed with general primer set 15F (AGAGTTTGATCCTGGCTCAG)/1492R (TACGGYTACCTTGTTACGACTT) and KOD-Plus-Neo polymerase (TOYOBO, Japan), using a thermal cycler with the following conditions: 95 °C for 5 min, 30 cycles of 98 °C for 10 s, 60 °C for 30 s, and 68 °C for 1 min.

The genome of mutant strains was sequenced using an Illumina HiSeq instrument (Illumina, San Diego, CA, USA). After the removal of adaptors, PCR primers, the content of N bases > 10%, and bases of quality lower than 20, clean data to the reference genome of strain ZM4 (GenBank No. NC_006526.2) was mapped. Annotation for potential SNVs were performed by Annovar (V21 Feb 2013). The sequencing was completed by GenWize Inc. (Suzhou, China).

## Conclusions

In this study, the adaptability of *Z. mobilis* in biogas slurry was enhanced via the ARTP mutagenesis. Several SNVs and InDels that are likely involved in tolerance to high concentration of biogas slurry and low pH were detected by genome re-sequencing. Moreover, biogas slurry was successfully used to replace water and nutrients to produce ethanol. The higher cell density were achieved via application of optimized medium in fed-batch fermentation, and the maximal value of ethanol titer, productivity, yields of theoretical reached 113.4 g/L, 4.13 g/L/h, 94.9%, respectively. After open fermentation, the maximal value of ethanol titer, productivity and yields of theoretical reached 108 g/L, 1.07 g/L/h and 89.9%, respectively. We concluded that S912 was a promising ethanol producer for biogas slurry fermentation. This strategy, replacing water and nutrients in ethanol production, not only reduce the cost in ethanol production, but also provide a valuable mean in the disposal of biogas slurry.

## Highlights

- The biogas slurry tolerant *Z. mobilis* strains were obtained via ARTP and ALE.
- Achieved an ethanol titer of 113.4 g/L after fed-batch fermentation.
- An effective strategy for ethanol production from biogas slurry was developed.

## Additional file

**Additional file 1.** Additional Figures and Tables.

## Data Availability

The *Z. mobilis* S912 and D95 have been deposited at Guangdong Microbial Culture Center (GDMCC) under the Accession Number GDMCC 60583 and GDMCC 60582, respectively.

## Abbreviations

AD: anaerobic digestion
ED: Entner–Doudoroff
CSTR: continuous stirred tank reactor
ARTP: atmospheric and room temperature plasma
ALE: adaptive laboratory evolution
RM: rich medium
OD: optical density
CDS: coding sequence
SNV: single-nucleotide variation
HPLC: high performance liquid chromatography
SV: structural variation
