# Supplementary material for: Replacing water and nutrients for ethanol production by ARTP derived biogas slurry tolerant Zymomonas mobilis strain
Source: Biotechnol Biofuels. 2019 May 20;12:124. doi: 10.1186/s13068-019-1463-2 (PMC6528197; doi:10.1186/s13068-019-1463-2)
Supplement: Supplementary file 1 — Additional file 1. Additional Figures and Tables. [file 13068_2019_1463_MOESM1_ESM.docx]

**Additional** **file**

Replacing Water and Nutrients for Ethanol Production by

ARTP Derived Biogas Slurry Tolerant Zymomonas mobilis Strain

Guowei Duan1, 2†, Bo Wu1†, Han Qin1, Weiting Wang1, 2, Qiong Tan1, 2,

Yonghua Dai1, 2, Yao Qin1, 3, Furong Tan1, 2, Guoquan Hu1, 2* and Mingxiong He1, 2*

1 Biomass Energy Technology Research Centre, Key Laboratory of Development and Application of Rural Renewable Energy (Ministry of Agriculture and Rural Affairs), Biogas Institute of Ministry of Agriculture and Rural Affairs, Section 4-13, Renmin Rd. South, Chengdu 610041, P. R. China.

2 Graduate School of Chinese Academy of Agricultural Science, Beijing 100081, P. R. China.

3 College of Pharmacy and Biological Engineering, Chengdu University, Chengdu 610041, P. R. China.

†These authors contributed equally to this work.

*Corresponding author (Fax: +86-28-85242281; E-mail: [huguoquan@caas.cn](mailto:huguoquan@caas.cn); hemingxiong@caas.cn)

**Additional Table 1** **Nutrient composition of biogas slurry**

| **Nutrient components** | **Contents (average ± SD, mg/L)** |
| --- | --- |
| Dissolvable Total Nitrogen (TN) | 1280±10 |
| Dissolvable Total Organic Carbon (COD) | 596±10 |
| NH_4_^+^-N | 670.06±10 |
| NO_3_^-^-N | 0.34±0.01 |
| Total Phosphorus (TP) | 144±1 |
| PO_4_^3-^-P | 61±8 |
| Potassium | 367±13 |
| Calcium | 102±12 |
| Sodium | 209±22 |
| Magnesium | 35±4 |
| Phosphorus | 163±8 |
| Sulfur | 709±18 |
| Iron | 0.79±0.18 |
| Zinc | 0.10±0.01 |
| Aluminium | 0.04±0.001 |
| Manganese | 2.06±0.37 |

**Additional Table 2 Conversion of glucose to ethanol by *Z. mobilis***

| **Strain** | **Fermentation**  **Time (h)** | **Glucose consumed**  **(g/L)** | **Ethanol** | | | |
| --- | --- | --- | --- | --- | --- | --- |
|  |  |  | **Titer**  **(g/L)** | **Yield**  **(g/g glucose)** | **Productivity**  **(g/L/h)** | **Theoretical**  **Yield (%)** |
| **RM, 50 g/L glucose，pH 6.0** | | | | | | |
| S912 | 24 | 50.2 | 23.4 | 0.47 | 0.98 | 92% |
| D95 | 24 | 50.3 | 23.6 | 0.47 | 0.98 | 92% |
| ZM4 | 31 | 50.7 | 23.4 | 0.46 | 0.75 | 90% |
| **Biogas slurry, 50 g/L glucose，pH 6.0** | | | | | | |
| S912 | 36 | 50.8 | 22.8 | 0.45 | 0.63 | 88% |
| D95 | 48 | 50.4 | 21.8 | 0.43 | 0.46 | 84% |
| ZM4 | 56 | 50.5 | 21.9 | 0.43 | 0.39 | 84% |

**Additional Table 3 Conversion of glucose to ethanol during fed‑batch fermentation**

| **Glucose addition** | **Fermentation**  **Time (h)** | **Glucose consumed**  **(g/L)** | **Ethanol** | | | |
| --- | --- | --- | --- | --- | --- | --- |
|  |  |  | **Titer**  **(g/L)** | **Yield**  **(g/g glucose)** | **Productivity**  **(g/L/h)** | **Theoretical Yield (%)** |
| **Sterilized，pH 6.0** | | | | | | |
| 1^st^ | 16 | 101.2 | 49.0 | 0.49 | 3.06 | 96% |
| 2^nd^ | 5 | 42.5 | 20.6 | 0.49 | 4.13 | 96% |
| 3^rd^ | 10 | 48.3 | 23.1 | 0.48 | 2.30 | 94% |
| 4^th^ | 10 | 50.2 | 21.9 | 0.44 | 2.19 | 86% |
| 5^th^ | 24 | 51.3 | 23.5 | 0.46 | 0.98 | 90% |
| **Unsterilized，pH 3.8** | | | | | | |
| 1^st^ | 108 | 100.2 | 46.0 | 0.46 | 0.43 | 90% |
| 2^nd^ | 48 | 73.9 | 33.1 | 0.45 | 1.07 | 88% |
| 3^rd^ | 56 | 83.6 | 36.8 | 0.44 | 0.67 | 86% |

**
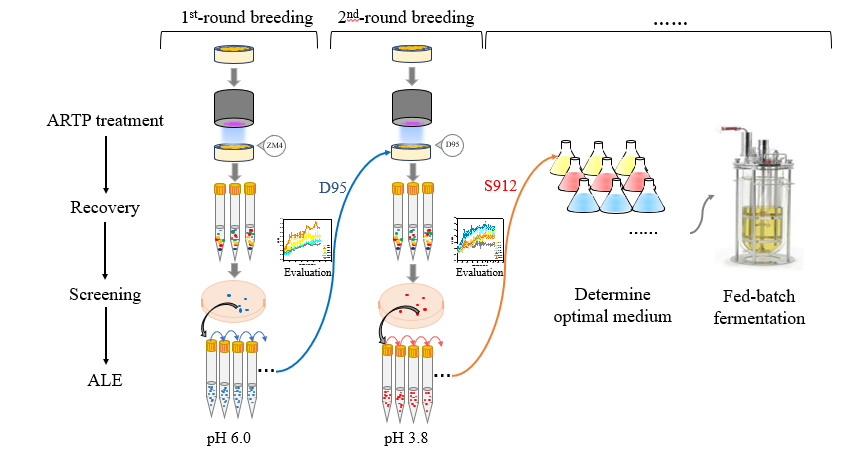
****Additional Figure. 1 Workflow for the strategy of ARTP mutagenesis compared with ALE.**

**
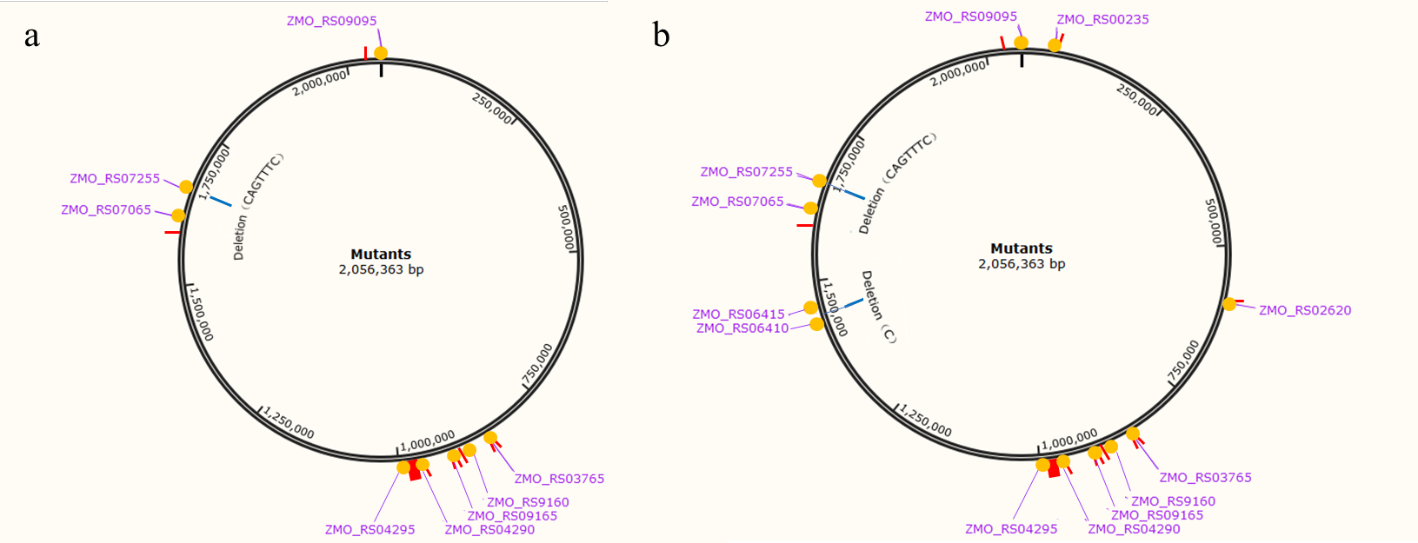
****Additional Figure. 2 The mutation sites in mutants compared with ZM4.** (a) the mutation sites of first-round mutants; (b) the mutation sites of second-round mutants. The red bars represent SNVs, and the blue bars represent deletions.
